# EXAMINING LIFE COURSE TRAJECTORIES WITH LINKED FULL COUNT IPUMS CENSUS DATA

**DOI:** 10.1093/geroni/igae098.1749

**Published:** 2024-12-31

**Authors:** Sarah Flood, Sarah Flood

**Affiliations:** Chair; Discussant

## Abstract

Understanding life course trajectories requires data with a long time horizon or sufficiently detailed retrospective recall measures; extending this to include intergenerational processes or mortality outcomes places further demands on data. Historical full count census data from IPUMS are a rich and underutilized source of information on early life conditions and can be harnessed to summarize millions life histories for individuals and trace families over multiple generations. IPUMS full count census data covers 1850-1950 and boast nearly 800 million individual records, which can be linked longitudinally across decennial censuses as well as to external data sources to answer questions about exposures and outcomes across the life course. This session will feature research presentations on the power of linked full count US census data to explore life course trajectories including substantive applications focused on racial equity in older-age mortality, negative economic shocks and long-term wellbeing, segregation and exposure to air pollution, and developing scores for estimating lifespan using commonly available socioeconomic and demographic measures. This session will highlight the value of historical census data to understanding current population and aging issues. The presentations will showcase research that links full count census data in multiple ways and demonstrates the flexibility and wealth of information available in these data.

